# Humus Acids in the Digested Sludge and Their Properties

**DOI:** 10.3390/ma15041475

**Published:** 2022-02-16

**Authors:** Anna M. Anielak, Aneta Kłeczek

**Affiliations:** Faculty of Environmental Engineering and Energy, Cracow University of Technology, Warszawska 24, 31-155 Krakow, Poland; aneta.kleczek@pk.edu.pl

**Keywords:** digested sludge, humus acids, fulvic acids, alpha (α) humic acids, hymatomelanic acids

## Abstract

Fulvic acids, alpha (α) humic acids and hymatomelanic acids were extracted digested sludge in two Cracow sewage treatment plants: Kujawy and Płaszów. Their elemental composition was examined and micropollution and ash content were determined. Based on the IR and UV-VIS spectrum, their similarities were determined with the occurring interactions with micropollution. Strong correlations between the acids coming from different sources depend on acid type and micropollution accompanying them, depending on concentration, influences to a specific extent their IR and UV-VIS spectra. Absorption analysis in infrared constitutes a simple method for characterizing fulvic and humic acids from wastewater treatment plants. The extracted fulvic acids were characterized by moderate maturity, while humus acids were well developed. In the fermentation process, the N bond increases together with the level of humification of the humus acid. The characteristics of the extracted humus acids comply with other humic substances presented in the literature. Quantitative analysis showed that digested sludge contains, on average: FA from 5.07 to 5.30 g/kg dry matter, αHA from 59.22 to 74.72 g/kg dry matter, HMA from 20.31 to 43.66 g/kg dry matter. It was thus demonstrated that wastewater treatment, in particular digested sludge, constitutes an attractive source of humus acids with a wide range of applications in numerous areas, such as agriculture, ecological rehabilitation, environmental protection, animal breeding, aquaculture, veterinary as well as medicine and is a precious source of soil fertilizers.

## 1. Introduction

The main components of humic substances (HS) are fulvic acids (FA) soluble within the entire pH range, humic acids (HA) soluble in alkaline solutions and precipitable at pH < 2 as well as insoluble humins (Hu). Hymatomelanic acid (HMA) is the HA fractions that are soluble in alcohol, the remaining part of HA is called alpha (α) humic acids (αHA). Humus acids (HAs) that contain C, N, O, H, S. Their elemental composition presented in different sources [1] depends on the place of their formation and extraction method. HAs contain numerous oxidized functional groups, such as hydroxyl and carboxyl groups representing strong chelation and adsorption [2,3,4]. In anaerobic conditions, they contribute to the reduction of methane emission [5], act as catalyzers in various dissolution and precipitation processes [6], e.g., in water and sewage they participate in dissolving pharmaceutical products [7] as well as other organic and inorganic substances, in the sorption and transport of metals [8], they form insoluble complexes with iron and manganese which significantly impedes the process of groundwater purification. HMA are pronounced capacity to cause antioxidant and antiradical effects [9]. HA has been found to have critical applications in many fields such as agriculture, ecological restoration, environmental protection, animal husbandry and aquaculture, veterinary medicine, human medicine [10,11,12,13,14,15,16]. HAs have a positive effect on the increase in total immunoglobulin and the functioning of the body [17]. FA slightly enhances cell viability and upregulates the protein, and most likely stimulates the immune-modulating molecules such as NO and induces cancer cell apoptosis [18]. The application of HAs in various areas is growing. At the same time, in Poland, nearly 65 thousand hectares of land require rehabilitation and renaturation. In Europe, degraded soils constitute 22.3% of their total surface. For this reason, it is important to obtain information on the sources of formation of humic substances. Until recently, it was claimed that HS is characteristic of surface water, groundwater and soil [19]. Their content in soil amounts to 85–90% of organic carbon [20] in natural water 60–80% [21], in seawater and water sludge ca. 10–80% of natural organic matter (NOM) [22].

HS affects soil properties, both physical and chemical, and improves its fertility. These substances improve soil properties like permeability and water retention capacity. They stimulate root-system development, which affects enzyme activity and increases the intake of nutrients [23]. Moreover, the water capacity is increased and the maintenance of a specific soil pH is supported by the presence of HS (buffer properties). HAs stimulate the growth of roots and leaf shoots, improving the quantity and quality of crops [24]. The sorption properties and ion exchange capacity of HS point to the possibility of using them to reduce soil salinity (limiting the concentration of Cl^−^ and NO_3_^−^), improve the efficiency of fertilisation (stabilisation of minerals, preventing flushing of groundwater) and detoxification. The immobilisation of heavy metals by HS reduces the bioavailability of metals to plants—it is similar in the process of removing heavy metals from wastewater, where HAs are used [23]. The water-retaining properties of humic substances result from their partially hydrophilic nature (the presence of hydroxyl and carboxyl groups) and porosity [25].

The research conducted in recent years demonstrates that an important source generating humic substances is waste management, in particular wastewater treatment plants and landfills [26,27,28,29,30,31,32,33,34]. An important source is constituted by digested sludge. In the process of methane fermentation, the humification of sewage sludge and humic substances contained in them takes place, which contributes to the formation of large quantities of HS at various levels of maturity; humification, aromatization, and carbonization. Very few studies have been carried out in a given research field. For this reason, it was decided to examine soluble HAs occurring in digested sludge and determine their properties and utility values.

## 2. Materials and Methods

### 2.1. Materials

For HA extraction, methane post-fermentation sludge from the “Płaszów” and “Kujawy” sewage treatment plants in Kraków was used, with different treatment efficiency. Developed sludge management is carried out in these plants. Preliminary sludge, fats picked from the surface of settlers, and excess sludge were directed to separated fermentation chambers (SFC). Digested sludge is thickened and dewatered in sedimentation centrifuges. For the purposes of the study, sludge before thickening and dewatering in centrifuges was applied. The characteristics of examined sludge from the Kujawy plant are presented in Table 1. The results demonstrate that it is highly hydrated (3% dry matter) and contains various metals, including heavy ones. It is the richest in potassium, ca. 10 g/kg dry matter, then zinc > 1 g/kg dry matter, barium ca. 230 mg/kg dry matter and manganese ca. 200 mg/kg dry matter. The TOC content is 55–62 g/kg dry matter, while Ba, Mn, Ag, Mg, Ca, As, Cr, Cd, Cu, Ni, Pb and Hg are present in trace quantities. Table 2 presents the composition of digested sludge from the Płaszów plant. The sludge contains more inorganic substances (4.2% dry matter), the remaining components are very similar to the values characteristic for digested sludge from the Kujawy plant. TOC content is 54–60.4 g/kg dry matter, potassium from ca. 10–18 g/kg dry matter, zinc from 1.2–1.3 g/kg dry matter. What is more, the sludge contains Ba, Mn, Ag, Mg, Ca, As, Cr, Cd, Cu, Ni, Pb and Hg. In general, digested sludge in both plants is characterized by a similar qualitative and quantitative composition, of major importance for the environment being the source of pollution in municipal wastewater.

### 2.2. Procedure of HAs Extraction and Analytic

**Scheme 1:**
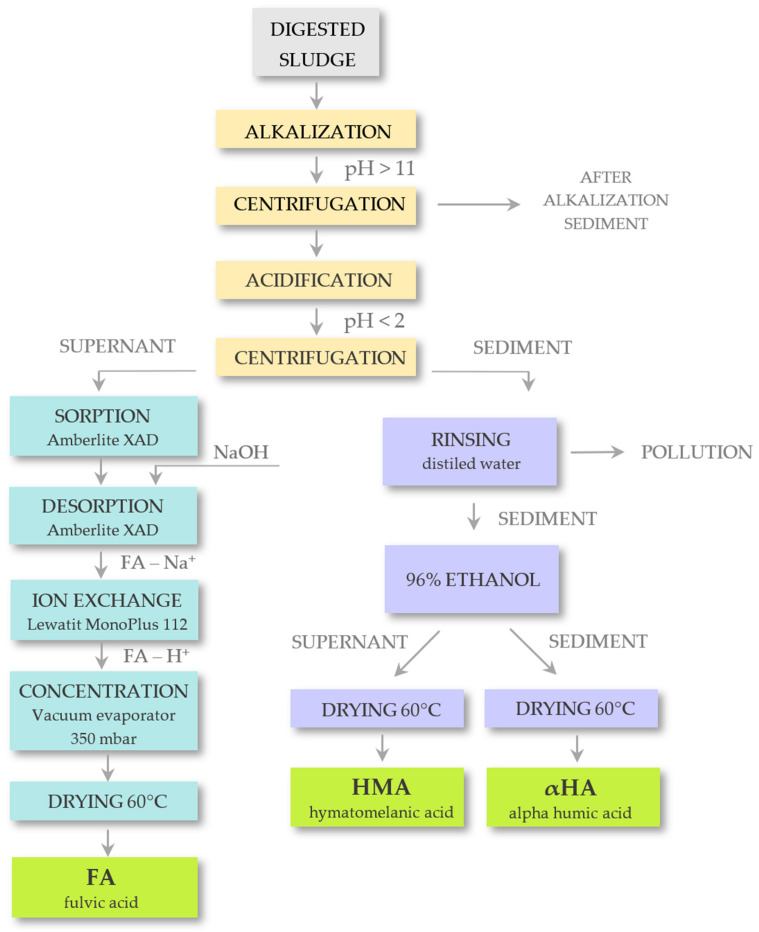
Technological system for extraction of humic substances from digested sludge.

We extracted from the digested sludge fulvic acids (FA), alpha (α) humic acid (αHA), and hymatomelanic acids (HMA) Scheme 1. The methodology used for extracting humus acids was in line with the recommendations of the International Humic Substances Society (IHSS), with a slight modification by the author.

The Amberlite XAD-1180 resins used were from Rohm and Haas (Philadelphia, PA, USA) and Lewatit MonoPlus 112(Lanxess, Delft, The Netherlands). HAs were extracted with 0.1 mol/L NaOH at room temperature for 24 h. The suspension was centrifuged at 15,000 G for 15 min. The alkaline extract contained HA (αHA, HMA) and FA.

The alkaline extract was acidified with hydrochloric acid (HCl A.C.S. 1:1) to a pH value of <2. After 24 h, this solution was centrifuged, and the supernatant containing FA was discarded. The sediment (which contained αHA and HMA) was repeatedly washed with distilled water. The solid residue was repeatedly treated with 96% ethanol to dissolve HMA and separate solid residue (contains αHA). Both fractions were lyophilised. The FA solution was passed through the Amberlite XAD-1180 with a speed of 1 L/h. The obtained desorbate was directed to a highly acidic cation exchanger (Lewatit MonoPlus 112) in the form of H^+^ [29]. The efflux was concentrated in a vacuum evaporator at a temperature of 60 °C and under the pressure of 350 mbar. The extracted HAs were subjected to qualitative analysis for the content of elements of elemental composition (C, H, N, O), inorganic impurities, including heavy metals, the IR spectrum and UV-VIS spectrum were determined, as well as ash content. The content of the basic elements is provided in the form of an ashless mass. The determination of carbon, hydrogen and nitrogen content was executed with the use of a combustion method with chromatographic detection (basic analysis). A 5–10 mg sample was used for the test. This determination was executed with an elementary analyser (Flash 2000 from Thermo Fisher Scientific, Waltham, MA, USA). The analysis of the remaining elements was done using X-ray fluorescence (XRF). The analysis was carried out in a powder analysis vessel on 4-μm-thick Proline film. Brune’s ED-XRF spectrometer, Bruker’s S8 Tiger spectrometer (Bruker AXS, Billerica, MA, USA), and Thermo’s EA Flash element analyzer were used. The ash designation was done by thermogravimetric method using the SDT Q600 Thermogravimeter from TA Instruments. The heating rate was 10 °C/min to 700 °C in the airflow of 100 mL/min. For the analysis, from 5 to 10 mg of sample was used.

Heavy metals in samples were determined with the use of the Inductively Coupled Plasma Mass Spectroscopy (ICP-MS) method, according to the standard test procedure, in compliance with PN-EN ISO 11885:2009. Infrared spectra was used for qualitative determination of humic substances. The identification was executed with the use of the Fourier-transform infrared spectroscopy (FTIR) method with the Attenuated Total Reflection (ATR) attachment. The spectral range was 450–4000 cm^−1^, with a resolution of 1 cm^−1^. For the purpose of determination, an FT-IR iS10 spectrometer Spectrum Two from PerkinElmer was used. The UV-VIS spectrum was determined on the WTW photoLab 7600 UV-VIS spectrophotometer (Xylem Analytics, Weilheim, Germany).

## 3. Results and Discussion

The characteristics of FA, αHA and HMA extracted from digested sludge in the Kujawy and Płaszów wastewater treatment plants in Cracow (Table 3) demonstrate their diversified composition. Carbon content in HMA from the Kujawy plant is low (26.56%), while the content of ash is high 51.90%, while αHA has the lowest content of inorganic pollution under the form of ash (3.5%). It is generally assumed that FA may contain carbon in the proportion of 38–54%, hydrogen 3–7%, nitrogen 0.6–9.5%, oxygen 36–52%, sulfur 0.5–0.7%. For HA the values range within 48–62% for carbon, 2.9–5.3% for hydrogen, 1.3–5.4% for nitrogen and 29.4–40.7% for oxygen. Extracted FA fall within the provided limits, while the content of αHA is more similar to HA. HMA is rich in nitrogen (10.35%), oxygen (56.08%). 

Based on the H/C and O/C values as well as the Krevelen’s diagram [35] it is possible to determine the type of HAs (FA or HA). The value of the fulvic acids quotient H/C (1.71) suggests their moderate maturity, also αHA for which H/C = 1.59. H/C values comply with the Krevelen’s diagram [35]. High H/C = 3.12 for HMA results from the presence of non-humic compounds in them. H/C atomic relations of pure humic substances [19] oscillate from 0.90 to 1.35 (there is on average one atom of hydrogen per one atom of carbon). This dependence has not been preserved here, in particular for HMA acids, for which the H/C quotient is high and amounts to 3.12. FA extracted from the sludge in Kujawy has the quotients of O/C = 0.71 and C/N = 12.29. O/C atomic relations (Table 3) represent the values compliant with those described by Tan et al. [19]. The acids from Kujawy and Płaszów are characterized by convergent O/C values, at the level of 0.71–0.73 for FA and 0.41–0.57 for αHA. O/C in the HMA from Płaszów equals 0.56. High O/C value, 1.58 for the acids from Kujawy points to the presence of non-humic pollution.

Nitrogen content in the studied αHA and HMA is about two to three times higher than in FA, suggesting that the N bond increases together with the level of humification of fulvic acids to humic acids. Growing quantities of nitrogen compounds are apparently sequestered in the process of HA molecule synthesis. The C/N quotient is the highest in FA. In αHA C/N = 7.91 and HMA C/N = 3.0. It is generally assumed that the C/N quotient will also decrease with the increase in the speed or extent of humification and C/N relationships between 10 and 15 are often considered characteristic for appropriately developed HAs [19]. An increase in the content of nitrogen in humus molecules demonstrates that, apart from the reaction of polymerization, for the purpose of including the necessary sources of nitrogen in a humus acid molecule, other reactions take place as well, such as interactions, sorption, chelation of nitrogen substances [19]. Elemental composition of acids extracted from digested sludge in the Płaszów plant (Table 3) is to a large extent compliant with the characteristics obtained for acids extracted from sludge in the Kujawy plant. Differences are observed in the content of carbon, with its highest value (48.06%) for αHA, similarly to acids extracted at the Kujawy plant. The highest content of ash was determined in HMA (41.32%), while the lowest in αHA (7.32%).

The characteristics of FA both when it comes to their elemental composition as well as atomic relations are the same in the samples from both plants, with the exception of the nitrogen content, which is slightly higher for the acids from Płaszów. The content of hydrogen and oxygen in FA is relatively high, which indicates their aliphatic nature. It is confirmed, for example, by a high H/C quotient (1.73) demonstrating the presence of developed aliphatic side chains in the structure of the examined FA. The extracted FA contain low quantities of nitrogen (C/N quotient equals 10.08).

αHA originating from the Płaszów plant are characterized by higher oxygen and ash content, resulting in higher O/C and O/H quotients compared with the samples from Kujawy. In turn, the C/N quotient is lower due to lower carbon content at comparable nitrogen content in the structure. According to generally adopted assumptions referring to the content of selected elements for subsequent humus acids, these values for the extracted FA fall within the limits presented above.

Elemental composition of HMA from Płaszów slightly differs from the composition of the same acids extracted in Kujawy. Increased carbon content is observed (45.42%), together with hydrogen (9.49%) and nitrogen (11.01%). Lower quantities of oxygen (34.08%) and ash (41.32%) are observed, which indicates their higher maturity. It is respectively confirmed by lower O/C and O/H quotients and higher C/N. O/C quotients for αHA and HMA indicate that in their molecule, there are two carbon atoms per one atom of oxygen. A high H/C quotient indicates the increase in the content of hydrogen, which results in an increased quantity of aliphatic structures. HMA is characterized by a low C/N quotient (4.80), which indicates high nitrogen content, and at the same time, it was observed that they contain more carbon and hydrogen than HMA from Kujawy and less oxygen.

**Figure 1:**
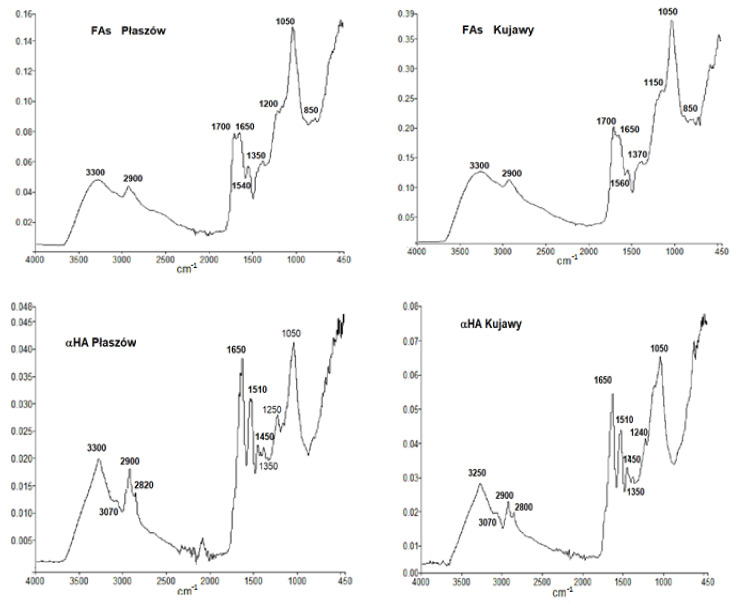

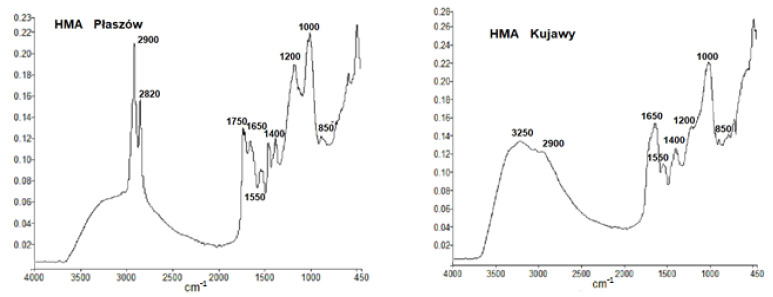
Infrared (FTIR) spectra HAs were recorded from digested sludge of the Płaszów plant: FA-Płaszów, αHA-Płaszów, HMA-Płaszów and form digested sludge of the Kujawy plant: FA-Kujawy, αHA-Kujawy, HMA-Kujawy.

The analysis of IR spectra presented in Figure 1 confirms the high level of similarity of the extracted HAs depending on the place of their occurrence. A particular analogy, not only in the type of the occurring absorption bands, but also in the general spectrum shape, can be observed for FA. The spectra of FA from Płaszów and Kujawy have the same bands: 850 cm^−1^, 1050 cm^−1^, 1150 cm^−1^, 1650 cm^−1^, 1700 cm^−1^, 2900 cm^−1^ and 3300 cm^−1^. Slight differences can be noticed for the bands 1350 cm^−1^ (Płaszów) and 1370 cm^−1^ (Kujawy) as well as for the bands 1540 cm^−1^ (Płaszów) and 1560 cm^−1^ (Kujawy) and they result from the presence of inorganic pollution in the acids.

The analysis of micropollution of HAs presented in Table 4 indicates that HAs from Płaszów, apart from the high content of chlorides, are characterized by a significant content of sodium, as follows for subsequent fractions: FA (18,400 mg/kg), αHA 16,100 mg/kg, relatively lower content of chlorides is characteristic for HMA (4400 mg/kg). A significantly lower content of sodium is observed in HS from Kujawy (from 20 to 4216). Phosphorus in the acids from Płaszów reaches the level of a dozen grams per 1 kg of the dry mass of the extracted acids: FA (16,300 mg/kg), HMA (13,100 mg/kg), and the content is the lowest for αHA (8500 mg/kg). The quantities of iron are slightly increased: FA (4300 mg/kg), HMA (900 mg/kg), αHA contain the most iron (10,500 mg/kg). Increased quantity of potassium 16,841 mg/kg is characteristic for HMA from Kujawy. The content of aluminum in the acids from Kujawy oscillates between 1083–1295 mg/kg and in the acids from Płaszów from 400 to 5700 mg/kg. The remaining elements, such as Zn, Mn, Cr, Mg, Ba, Sr, Si, Cu, Ni, Ag are not observed or are present in trace quantities.

The bands 1650 cm^−1^ and strong ones at 1050 cm^−1^ are attributed to the vibrations of OH groups; aliphatic C–H, carbonyl (C=O), carboxyl (COO); and respectively ethyl, vinyl (CH–CH_2_), aromatic with aldehyde, amino and thiol group (SH). The similarity of FA bands of the acids originating from different sources (Kujawy, Płaszów) is surprising and indicates that the analysis of the IR spectrum for the identification of subsequent acids constitutes the correct method. The bands 2900 cm^−1^ and 3300 cm^−1^ represent the content of aliphatic groups, the band 1700 cm^−1^ of carboxylic acid groups, 1540–1560 cm^−1^ probably protein material, while 1150 cm^−1^ and 1050 cm^−1^ represent the lignin-like material. A high level of similarity is also observed for the bands αHA Płaszów and αHA Kujawy. It is indicated by common bands 1050 cm^−1^, 1350 cm^−1^, 1450 cm^−1^, 1510 cm^−1^, 1650 cm^−1^, 2900 cm^−1^, 3070 cm^−1^ and with slight deviation the bands 1240 cm^−1^ (Kujawy) and 1250 cm^−1^ (Płaszów) as well as 2800 cm^−1^ (Kujawy) and 2820 cm^−1^ (Płaszów), which shall be explained by the presence of micropollution. The αHA spectra (Kujawy, Płaszów) are characterized by strong aliphatic absorption band C–H between 2900 cm^−1^ and 3250–3300 cm^−1^. The 1650 cm^−1^ band is responsible for absorption in COO carboxyl groups. Absorption analysis in infrared thus constitutes the easiest method of characterizing fulvic and humic acids originating from a wastewater treatment plant.

Slight dislocations of bands (e.g., Płaszów 1200, 1350, 1540; for Kujawy 1150, 1370, 1370) result from the presence of inorganic micropollution, FA from Płaszów contain 14.66% of ash ad from Kujawy 17.29%. The lowest diversification was obtained for αHA characterized by low ash content (αHA from Płaszów 7.32%, αHA from Kujawy 3.50%). When it comes to HMA, characterized by high ash content (Płaszów 41.32%, Kujawy 51.90%), diversified absorption is observed in infrared. Apart from joint bands 1000 cm^−1^, 1200 cm^−1^, 1400 cm^−1^, 1550 cm^−1^, 1650 cm^−1^ and 2900 cm^−1^, HMA from Płaszów contain 1750 cm^−1^ and 2820 cm^−1^, HMA from Kujawy 3250 cm^−1^.

**Figure 2:**
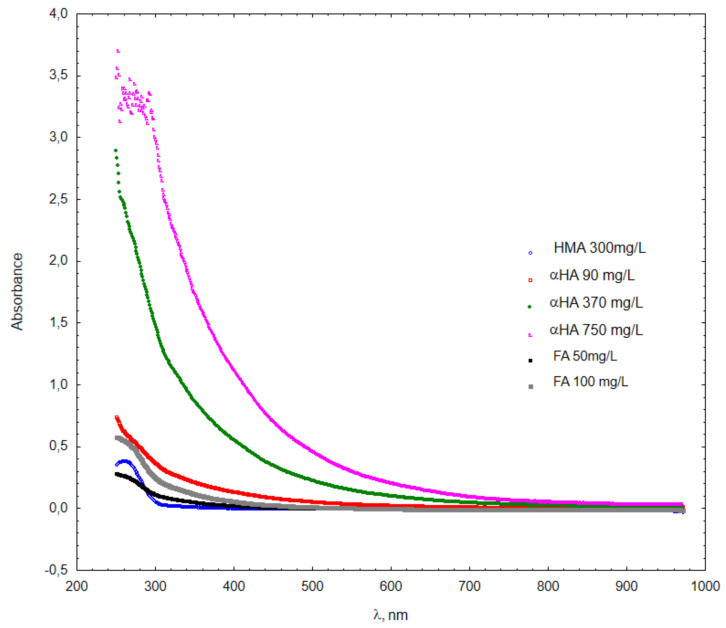
UV-VIS spectra of humus acids extracted from digested sludge of the Kujawy wastewater treatment plant.

**Figure 3:**
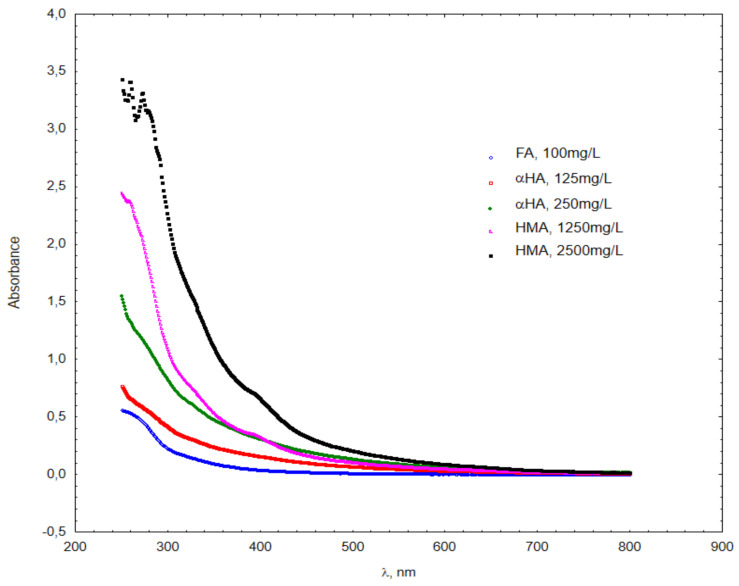
UV-VIS spectra of humus acids extracted from digested sludge of the Płaszów wastewater treatment plant.

Optical properties in the UV-Vis light constitute another characteristic of HAs. Basing on UV-Vis spectra within the range 250–780 nm (Figure 2 and Figure 3) specified for subsequently extracted acids at their various concentrations for selected wavelength λ nm, the quotients of absorbance values A_250_/A_465_ and A_250_/A_350_ were calculated. Absorption curves within the VIS range indicate the monotonous character of the increase in the light absorbance value together with the increasing value for wavelength. The value of the A2/A4 ratio (absorbance quotient for the light with wavelength 250 and 465 nm) constitutes the indicator of the quantity of organic substance at the initial decomposition stage, A2/A6 informs about the presence of substances resistant to humification, such as lignin for example. 

Together with the increase in condensation and aromatization of the molecules of humus acids, the A4/A6 value decreases and the C/H quotient grows. The A2/A4 quotient is responsible for the size and aromaticity of molecules [36]. A2/A4 quotients for FA are very similar (Kujawy 32.3; Płaszów 33), as it is also the case for αHA (Kujawy 9.79; Płaszów 8.8), disproportions are observed for HMA (Kujawy 121; Płaszów 15.5) that contain large quantity of ash. Similar values were obtained for the A2/A3 quotient for αHA acids (Kujawy 3.48; Płaszów 3.31), FA (Kujawy 5.20; Płaszów 5.91) as well as discrepancy for HMA (Kujawy 24.13; Płaszów 3.93). Relatively low quotients A4/A6 for acids from the Płaszów plant (FA 8.50; αHA 4.67 and HMA 5.58) indicate a high level of condensation and aromatization of HAs molecules. Basing on given indicators it is possible to state that the extracted FA and HA represent the same level of aromatization and condensation as well as the size and aromaticity of their molecules. The absence of such an analogy for HMA results from significant ash content. The characteristics of acids extracted from sludge in different wastewater treatment plants are similar and specific for acid type. Curve deflection at the wavelength of 270 nm indicates the presence of aromatic rings in an acid molecule.

Quantities of the extracted acids per kg dry matter of digested sludge (Table 5) demonstrate that digested sludge constitutes a rich source for obtaining humic substances. It is possible to extract ca. 100 g HAs from 1 kg dry matter of digested sludge.

**Figure 4:**
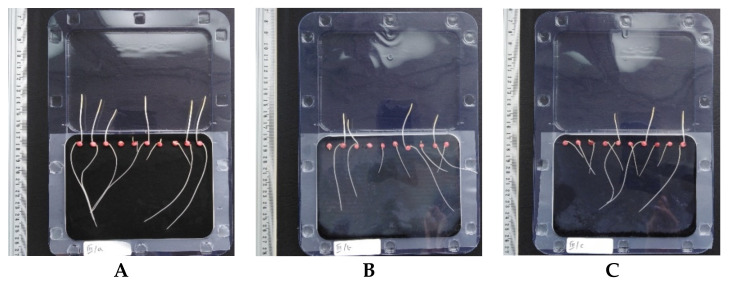
Effect of HAs, nitrogen fertilizer and phosphorus on the growth of *Sorghum saccharatum*. (**A**) Has. (**B**) nitrogen fertilizer. (**C**) phosphorus fertilizer.

Research using the Phytotoxkit tests and with three tested plants, *Sorghum saccharatum*, *Lepidium sativum*, and *Sinapis alba*, showed that the plants thrive best in the presence of HAs. The extracted humic substances from digested sludge provide a good substrate for the growth of the plants in question. HAs turned out to be competitive in relation to synthetic phosphorus and nitrogen fertilizers. Figure 4 shows selected test results confirming the useful nature of HAs contained in the digested sludge. The roots and shoots of *Sorghum saccharatum* thrives best in the presence of HAs.

Detailed research in this area is currently underway. A simple and economically viable technology is also being developed to extract humic substances from fermented sludge.

## 4. Conclusions

Digested sludge contains large quantities of HAs, ca. 100 g/kg dry matter. Their qualitative characteristics are similar irrespective of the source of their occurrence. Within the humification process of FA to HA, visible strengthening of the N bond takes place in αHA and HMA acids. These substances are rich in carbon (FA ca. 45%, αHA) and their composition is favorable for the soil. 

For this reason, digested sludge constitutes an attractive source for obtaining humic substances, which is important in view of the global trend of soil depletion and degradation. At the same time, leachates recirculated to biological reactor within the wastewater treatment plant contribute to the occurrence of harmful struvite, and due to the significant content of humic substances resistant to biodegradation, they hinder the wastewater treatment process. Research with Phytotoxkit tests has shown that the extracted HAs can be used as a soil amendment and have better properties than synthetic phosphorus and nitrogen fertilizers. The greatest efficiency of plant growth is obtained using a compilation of HAs and phosphorus fertilizer. However, the method of their isolation should be simple, and it should eliminate parasites and other pathogenic microorganisms occurring in sewage sludge. Therefore, research should be continued towards simplifying the technology of extracting humic substances, which will have a positive effect on the economic factor, which is very important in the case of mass use of the product.

Independently, studies have shown a close correlation in the characteristics of subsequent FA, HA (αHA, HMA) from digested sludge originating from different wastewater treatment plants. The content of ash may, to some extent, influence the deviations in IR and UV-VIS absorption bands.

## Figures and Tables

**Table 1:** Characteristics of digested sludge from a separate fermentation chamber at the Kujawy sewage treatment plant, the dry matter content in the sludge is 3% (Source: Project Nr POIR.04.01.04-00-0039/17).

KUJAWY Sludge	Ba	Mn	TOC	K	Ag	Mg	Ca	As	Cr	Cd	Cu	Ni	Pb	Hg	Zn
	mg/kg DM	mg/kg DM	mg/kg DM	mg/kg DM	mg/kg DM	% DM	% DM	mg/kg DM	mg/kg DM	mg/kg DM	mg/kg DM	mg/kg DM	mg/kg DM	mg/kg DM	mg/kg DM
Sample 1	228	197	57,428	10,599	<10	0.315	6.17	<10	309	<10	233	33.4	15.8	0.845	1151
Sample 2	235	208	62,841	10,467	<10	2.260	6.05	<10	323	<10	241	34.9	16.8	0.908	1194
Sample 3	233	205	55,887	9981	<10	1.920	6.88	<10	304	<10	237	35.0	18.4	1.010	1200
Sample 4	232	201	60,752	10,348	<10	1.490	6.30	<10	310	<10	220	34.4	16.6	0.624	1184
Avg.	232	203	59,227	10,349	<10	1.496	6.35	<10	312	<10	233	34.4	16.9	0.847	1182

DM—dry matter.

**Table 2:** Characteristics of digested sludge from a separate fermentation chamber at the Płaszów sewage treatment plant, the dry matter content in the sludge is 4.2%. (Source: Project Nr POIR.04.01.04-00-0039/17).

PŁASZÓW Sludge	Ba	Mn	TOC	K	Ag	Mg	Ca	As	Cr	Cd	Cu	Ni	Pb	Hg	Zn
	mg/kg DM	mg/kg DM	mg/kg DM	mg/kg DM	mg/kg DM	% DM	% DM	mg/kg DM	mg/kg DM	mg/kg DM	mg/kg DM	mg/kg DM	mg/kg DM	mg/kg DM	mg/kg DM
Sample 1	245	232	54,592	10,016	<10	1.84	5.05	<10	161	<10	276	69.6	34.1	0.699	1274
Sample 2	244	237	59,892	11,596	<10	2.05	4.90	<10	159	<10	273	68.3	34.2	0.457	1264
Sample 3	238	221	60,393	17,936	<10	3.40	3.44	<10	154	<10	274	67.5	32.5	0.466	1300
Avg.	242	230	58,292	13,183	<10	2.43	4.46	<10	158	<10	274	68.47	33.6	0.541	1279

DM—dry matter.

**Table 3:** Elemental composition and its atomic ration for the extracted humus acids. Kujawy & Płaszów.

Samples	Composition Characteristic Humus Acids								
	Elementals Content Ash Free (%)				Atomic Ration (-)				Ash Contents (%)
Kujawy	C	O	H	N	O/C	H/C	C/N	O/H	
FA	45.69	43.42	6.53	4.36	0.71	1.71	12.29	0.42	17.29
αHA	54.70	29.97	7.27	8.06	0.41	1.59	7.91	0.26	3.50
HMA	26.65	56.08	6.92	10.35	1.58	3.12	3.00	0.51	51.90
Płaszów									
FA	44.83	43.47	6.47	5.23	0.73	1.73	10.08	0.42	14.66
αHA	48.06	36.46	7.13	8.35	0.57	1.79	6.68	0.32	7.32
HMA	45.42	34.08	9.49	11.01	0.56	2.50	4.80	0.22	41.32

**Table 4:** Micropollutants of humus acids extracted from digested sludge from the Kujawy and Płaszów wastewater treatment plants.

Ion	Kujawy			Płaszów		
	FA mg/kgDM	αHA mg/kgDM	HMA mg/kgDM	FA mg/kgDM	αHA mg/kgDM	HMA mg/kgDM
Cl	-	-	-	60,800	31,900	90,800
Na	285	20	4216	18,400	16,100	4400
P	-	-	-	16,300	8500	13,100
Fe	589	5264	1494	4300	10,500	900
Ca	436	127	2477	800	6000	378
Br	-	-	-	500	-	-
Al	1151	1293	1083	400	5 700	423
K	252	145	16,841	84	2500	2700
Zn	78	428	956	79	400	1900
Mn	9	51	24	39	3	12
Cr	22	70	18	21	31	11
Mg	45	87	336	5	161	664
Ba	8	1	13	4	17	-
Sr	-	-	-	2	13	-
Si	-	-	-	-	2100	-
Cu	27	281	47	-	242	800
Ni	20	-	21	-	-	81
Ag	-	20	-	-	-	6

**Table 5:** Quantity of the extracted acids.

Humus Acids	Płaszów (g/kg DM)	Kujawy (g/kg DM)
FA	5.07	5.29
αHA	74.72	59.22
HMA	43.66	20.31

## Data Availability

Cracow University of Technology Faculty of EEE Project report number POIR.04.01.04-00-0039/17.

## References

[1] Machado W., Franchini J.C., de Fátima Guimarães M., Filho J.T. (2020). Spectroscopic characterization of humic and fulvic acids in soil aggregates. Heliyon.

[2] Chua S.F., Nouri A., Ang W.L., Mahmoudi E., Ba-Abbad M. (2021). The emergence of multifunctional adsorbents and their role in environmental remediation. J. Environ. Chem. Eng..

[3] Satisha G.C., Devarajan L. (2005). Humic substances and their complexation with phosphorus and calcium during composting of pressmud and other biodegradables. Commun. Soil Sci. Plant Anal..

[4] Gong G.Q., Zhao Y.F., Zhang Y.J., Deng B., Liu W.X., Wang M., Yuan X., Xu L.W. (2020). Establishment of a molecular structure model for classified products of coal-based fulvic acid. Fuel.

[5] Tan W., Jia Y., Huang C., Zhang H., Li D., Zhao X., Wang G., Jiang J., Xi B. (2018). Increased suppression of methane production by humic substances in response to warming in anoxic environments. J. Environ. Manag..

[6] Zashikhina A.V., Sviridova M.L. (2019). Gold Leaching with Humic Substances. J. Min. Sci..

[7] Khan R., Jain P., Aqil M., Agarwal S.P., Mirza M.A., Iqbal Z. (2020). Pharmacokinetic evaluation of fulvic acid-ketoconazole complexes: A validation and line extension study. J. Drug Deliv. Sci. Technol..

[8] Mou J., Li C., Wang G., Shi W. (2012). Complexation and sorption studies of Co(II) with [gamma]-alumina-bound fulvic acid: Effect of pH, ionic strength, fulvic acid and alumina concentration. J. Radioanal. Nucl. Chem..

[9] Efimova I.V., Khil’ko S.L., Smirnova O.V., Berezhnoi V.S., Rybachenko V.I. (2013). Antioxidant properties of hymatomelanic acids from brown coal. Solid Fuel Chem..

[10] Kapoore R.V., Wood E.E., Llewellyn C.A. (2021). Algae biostimulants: A critical look at microalgal biostimulants for sustainable agricultural practices. Biotechnol. Adv..

[11] Xu D., Deng Y., Xi P., Yu G., Wang Q., Zeng Q., Jiang Z., Gao L. (2019). Fulvic acid-induced disease resistance to Botrytis cinerea in table grapes may be mediated by regulating phenylpropanoid metabolism. Food Chem..

[12] Ge X.F., Wang L.J., Zhang W.J., Putnis C.V. (2020). Molecular Understanding of Humic Acid-Limited Phosphate Precipitation and Transformation. Environ. Sci. Technol..

[13] Fizer M., Sidey V., Milyovich S., Fizer O. (2020). DFT study of fulvic acid binding with bivalent metals: Cd, Cu, Mg, Ni, Pb, Zn. J. Mol. Graph..

[14] Qin Y., Zhu H., Zhang M., Zhang H., Xiang C., Li B. (2016). GC-MS Analysis of Membrane-Graded Fulvic Acid and Its Activity on Promoting Wheat Seed Germination. Molecules.

[15] Abdel-Baky Y.R., Abouziena H.F., Amin A.A., El-Sh M.R., El-Sttar A.M.A. (2019). Improve quality and productivity of some faba bean cultivars with foliar application of fulvic acid. Bull. Natl. Res. Cent..

[16] Gong G., Yuan X., Zhang Y., Li Y., Liu W., Wang M., Zhao Y., Xu L. (2020). Characterization of coal-based fulvic acid and the construction of a fulvic acid molecular model. RSC Adv..

[17] Morales O.Y., Navarrete J.M., Gracia I., Rivera M., Sánchez F. (2014). Fulvic acids, fertilizers first and then stimulants of vital functions in vertebrates. J. Radioanal. Nucl. Chem..

[18] Jayasooriya R., Dilshara M.G., Kang C.H., Lee S., Choi Y.H., Jeong Y.K., Kim G.Y. (2016). Fulvic acid promotes extracellular anti-cancer mediators from RAW 264.7 cells, causing to cancer cell death in vitro. Int. Immunopharmacol..

[19] Tan K.H. (2014). Humic Matter in Soil and the Environment: Principles and Controversies.

[20] Peng A., Xu Y., Wang Z.J. (2001). The effect of fulvic acid on the dose effect of selenite on the growth of wheat. Biol. Trace Elem. Res..

[21] Boult S., Jugdaohsingh R., White K., Smith B., Powell J. (2001). Evidence that polysaccharide and humic and fulvic acids are co-extracted during analysis but have different roles in the chemistry of natural waters. J. Appl. Geochem..

[22] Rodriguez F.J., García-Valverde M. (2016). Influence of preozonation on the adsorptivity of humic substances onto activated carbon. Environ. Sci. Pollut. Res..

[23] Yang F., Antonietti M. (2020). The sleeping giant: A polymer View on humic matter in synthesis and applications. Prog. Polym. Sci..

[24] Hassan M.K., McInroy J.A., Kloepper J.W. (2019). The Interactions of Rhizodeposits with Plant Growth-Promoting Rhizobacteria in the Rhizosphere: A Review. Agriculture.

[25] Jaeger F., Shchegolikhina A., van As H., Schaumann G.E. (2010). Proton NMR Relaxometry as a Useful Tool to Evaluate Swelling Processes in Peat Soils. J. Magn. Reson..

[26] Yang Y., Li H., Li J. (2014). Variation in humic and fulvic acids during thermal sludge treatment assessed by size fractionation, elementary analysis, and spectroscopic methods. Front. Sci. Eng..

[27] Li H., Li Y., Li C. (2017). Evolution of humic substances during anaerobic sludge digestion. Environ. Eng. Manag. J..

[28] Li H., Li Y., Zou S., Li C. (2014). Extracting humic acids from digested sludge by alkaline treatment and ultrafiltration. J. Mater. Cycles Waste Manag..

[29] Anielak A.M., Świderska R. (2001). The influence of the adsorbents’ electrokinetic potential on the adsorption process of humic substances. Environ. Prot. Eng..

[30] Łomińska-Płatek D., Anielak A.M. (2021). Quantitative balance and analysis of fulvic acids changes in the process of municipal sewage treatment. Water Resour. Ind..

[31] Orliński T., Anielak A.M. (2021). Characteristics of fulvic acids generated in communes waste landfills. Arch. Environ. Prot..

[32] Xiaoli C., Shimaoka T., Qiang G., Youcai Z. (2008). Characterization of humic and fulvic acids extracted from landfill by elemental composition, ^13^C CP/MAS NMR and TMAH-Py-GC/MS. Waste Manag..

[33] Xiaoli C., Guixiang L., Xin Z., Yongxia H., Youcai Z. (2012). Fluorescence excitation–emission matrix combined with regional integration analysis to characterize the composition and transformation of humic and fulvic acids from landfill at different stabilization stages. Waste Manag..

[34] Usmani Z., Sharma M., Awasthi A.K., Sivakumar N., Lukk T., Pecoraro L., Gupta V.K. (2021). Bioprocessing of waste biomass for sustainable product development and minimizing environmental impact. Bioresour Technol..

[35] Bai Y., Wu F., Xing B., Meng W., Shi G., Ma Y., Giesy J.P. (2015). Isolation and Characterization of Chinese Standard Fulvic Acid Sub-fractions Separated from Forest Soil by Stepwise Elution with Pyrophosphate Buffer. Sci. Rep..

[36] Trubetskaya O.E., Trubetskoj O.A., Voyard G., Richard C. (2013). Determination of hydrophobicity and optical properties of soil humic acids isolated by different methods. J. Geochem. Explor..

